# Do Binding Moral Foundations Predict a Hypothetical Moral Behavior? The Moderating Role of the Perception of the In-Group Moral Standards

**DOI:** 10.3390/bs15030265

**Published:** 2025-02-24

**Authors:** Ankica Kosic, Annalisa Theodorou, Luigi Leone

**Affiliations:** 1 Faculty of Medicine & Psychology, Sapienza University of Rome, 00185 Rome, Italy; luigi.leone@uniroma1.it; 2 Department of Systems Medicine, Tor Vergata University of Rome, 00133 Rome, Italy; annalisa.theodorou@uniroma2.it

**Keywords:** moral foundations, binding foundations, moral behavior, moral dilemmas, social norms

## Abstract

Previous studies have found a positive relationship between binding moral foundations and negative inter-group attitudes. Nevertheless, some studies have shown that, under specific conditions, binding foundations can also lead to positive outcomes, particularly within the intra-group context. In this research, we hypothesize that when people perceive that some in-group members violate moral norms, individuals with stronger binding moral foundations may exhibit a greater preference for moral choices in hypothetical moral dilemmas. This hypothesis was confirmed in Study 1 (N = 184) and replicated in Study 2 (N = 201), both conducted in Italy. In Study 1, we utilized a questionnaire containing the moral foundation questionnaire, while in Study 2, we employed moral foundation vignettes. In both studies, participants were presented with five scenarios describing hypothetical moral dilemmas that could occur in real-life settings. The findings indicate that binding moral foundations can lead to stronger preferences for moral choices in hypothetical moral dilemmas when the morality of the in-group is perceived to be under threat. These results are discussed in light of their implications for future research on binding foundations.

## 1. Introduction

The study of morality has accompanied the history of humankind. But what explains and predicts moral behaviors? Various theories have been proposed, initially in philosophy and, more recently, in psychology. One of the most influential psychological frameworks addressing morality is the Moral Foundations Theory (MFT; Haidt, 2007, 2008; Haidt & Graham, 2007; Haidt et al., 2009; Haidt & Joseph, 2004). This theory suggests that moral behavior is guided by at least five foundational principles that serve as the basis for moral judgments and actions. These foundations are observed across cultures and are considered innate, resulting from evolutionary adaptation (Haidt, 2013). Haidt (2013) explained that these foundations are not entirely fixed; they can be shaped to some extent by contextual factors, leading to cultural variations in how they manifest (see also Atari et al., 2022; Haidt & Graham, 2007; Shweder et al., 1997). Indeed, the observation that every culture has distinct views on morality and immorality, as well as on what is considered right or wrong, suggests that morality is deeply influenced by cultural context (Prinz, 2008). Morality is shared within a group, which defines moral norms and establishes ideas about what is considered moral (for a general discussion, see Bar-Tal, 2000; Leach et al., 2015). Thus, groups serve as the locus of moral values and act as our moral compass. Individuals tend to align their behavior with the norms established by their groups and are often regulated by these norms. Therefore, understanding the types of groups we belong to and how they are perceived in terms of morality is crucial.

This study has three main objectives, which are as follows: (1) to explore how individuals make choices in hypothetical moral dilemmas and whether these choices are influenced by their perception of the in-group’s moral standards and their own moral foundations; (2) to investigate how individuals respond to hypothetical moral dilemmas when they perceive that their in-group holds low moral standards; and (3) to examine whether binding moral foundations can be considered sources of morality, as proposed by Haidt and collaborators (e.g., Haidt & Joseph, 2008), a claim that has been questioned by other scholars (e.g., Gray & Keeney, 2015; Kugler et al., 2014; Musschenga, 2013).

### 1.1. The Importance of the In-Group’s Morality

Various studies have demonstrated that morality plays a crucial role in shaping social impressions (e.g., Brambilla et al., 2011, 2012; Wojciszke, 2005; Wojciszke et al., 1998). The existence of moral codes within groups enables individuals to develop an understanding of what is considered moral (Bar-Tal, 2000; Ellemers & van den Bos, 2012). In-groups facilitate this process by allowing individuals to compare themselves with other members or align with group norms (e.g., Leach & Vliek, 2008).

Leach et al. (2007) confirmed that identification with an in-group is stronger when the group is perceived as highly moral (see also Theodorou & Kosic, 2021). People are motivated to define their groups as moral because belonging to a dishonest group can result in significant costs, including negative identity and low self-esteem (Ellemers et al., 2013, 2008; Leach et al., 2007; Pagliaro et al., 2011, 2016). When individuals identify with a group, they tend to adopt the group’s moral norms and use these norms as a framework for interpreting the social world (Ellemers et al., 2008). Haidt and collaborators proposed a similar perspective but approached it from a more functional rather than normative angle, emphasizing the role of group norms in regulating moral behavior and group dynamics (Haidt & Joseph, 2008).

### 1.2. The Moral Foundations Theory

The moral foundations theory (MFT) identifies the following five (or six) moral foundations: care, fairness, authority, loyalty, purity, and liberty (proposed as a potential sixth; Haidt & Joseph, 2008).

Care/Harm: This foundation originates from the need to care for and protect children. The virtues and emotions associated with this foundation include caring, kindness, and compassion.

Fairness/Cheating: This foundation arises from the need for mutual collaboration. Relevant emotions include gratitude and guilt (for upholding or violating fairness) and anger in the case of violations. Associated virtues are fairness, justice, and trustworthiness.

These two foundations are collectively referred to as individualizing foundations due to their emphasis on the well-being of individuals. They encourage people to respect others’ rights, assist those in need, and treat others fairly, although they also have broader societal and group-level implications.

To address aspects of morality related to societal survival and coexistence, Haidt and colleagues propose the following three additional foundations:

Loyalty/Betrayal: This foundation stems from the human need to belong to groups. It is triggered by threats to the group and is associated with virtues such as loyalty, respect for traditions, patriotism, commitment, sacrifice, and vigilance against betrayal. Emotions connected to this foundation include group pride and anger toward deviants.

Authority/Respect vs. Subversion: This foundation emphasizes respect for authority, rules, and institutions. It is activated when individuals perceive disrespect, disobedience, or non-conformity (Graham et al., 2013). Violations elicit feelings of contempt.

Purity/Sanctity vs. Degradation: This foundation focuses on preserving the dignity, purity, cleanliness, and sacredness of the in-group. It is triggered by perceived threats to the group’s essence, as well as its cultural or religious identity. The primary emotion in response to transgressions is disgust, and associated virtues include temperance, chastity, wholesomeness, and the control of desires.

These three dimensions are often referred to as binding foundations because they emphasize the importance of social cohesion and maintaining group integrity.

The binding foundations serve to encourage behaviors that protect the in-group and prohibit behaviors that destabilize it. These foundations emphasize the importance of group solidarity, respect for authority, and purity. To safeguard these values, people establish well-defined roles within in-groups and create institutional systems to maintain order and cohesion (Federico et al., 2013). The binding moral foundations provide “external guidelines for a moral character” (Federico et al., 2016, p. 228) and “regulate individual selfishness and make social life possible” (Haidt, 2008, p. 79).

The moral foundations questionnaire (MFQ30) was proposed by Graham et al. (2008) and has been validated in hundreds of studies across various countries. Psychometric evaluations, however, yield contradictory results regarding the structure of moral foundations, supporting either the five-factor model (basic foundations) or the two-factor model (individualizing and binding). Some studies favor the five-factor model (e.g., Nilsson & Erlandsson, 2015; Yilmaz et al., 2016), while others support the two-factor model (e.g., Harper & Rhodes, 2021; Graham et al., 2011; Lewis & Bates, 2010; K. B. Smith et al., 2017). More recently, however, findings on the factorial structure of the MFQ have reinforced the theoretical link between the two-factor model and the individualizing/binding distinction (Harper & Rhodes, 2021).

Neuropsychological studies further support this distinction by identifying different anatomical regions associated with the individualizing and binding moral foundations (e.g., Lewis et al., 2012). These findings suggest a commonality among the five foundations that aligns with a broader two-factor structure. In addition to statistical contradictions, some authors have raised conceptual concerns regarding the individualizing/binding distinction. For instance, it has been argued that fairness is not strictly focused on the individual and that purity is not solely associated with the group (e.g., Janoff-Bulman & Carnes, 2013). Although the MFQ exhibits important psychometric limitations, there is no compelling reason to dismiss the two-factor model entirely.

### 1.3. The Binding Moral Foundations

Throughout history, principles such as obedience, loyalty, and purity have been crucial in uniting people and enabling them to thrive as groups, tribes, and nations. However, these same principles have also contributed to in-group favoritism, inter-group conflicts, and even genocide. Consequently, several authors have questioned the classification of binding foundations as dimensions of morality (e.g., Kugler et al., 2014; Musschenga, 2013). Research has consistently shown that individuals who strongly endorse binding values tend to have a more conservative political orientation (e.g., Wright & Baril, 2011). Baldner and Pierro (2019) found that binding foundations predict prejudice toward migrants, while Kugler et al. (2014) linked binding to homophobia, sexism, authoritarianism, and in-group favoritism. Additionally, Mededović and Petrović (2016) demonstrated that binding foundations are associated with the dark triad personality traits. These findings challenge the theoretical assumptions of moral foundations theory (MFT) regarding the moral nature of binding foundations. Despite these studies, Haidt has repeatedly emphasized that binding foundations are rooted in moral values and do not always lead to negative outcomes (Haidt, 2016). Some research has identified conditions under which the endorsement of binding moral foundations can result in positive outcomes. For example, Malka and colleagues (Malka et al., 2016) found that binding foundations are associated with lower levels of ideological polarization on political issues that lack inherent relevance to moral traditionalism. Our research challenges the assumption that binding foundations fall outside the realm of morality. Overall, we argue that concepts such as the respect for authority and loyalty are not inherently negative. While they can sometimes lead to undesirable outcomes, such as discriminatory in-group bias and out-group derogation, we contend that under certain conditions, binding foundations serve as moral guidelines, particularly within the in-group.

### 1.4. Research Overview

We predict that participants with a strong endorsement of binding foundations will exhibit more moral behavior in hypothetical moral dilemmas, particularly when they perceive their in-group as violating moral standards or norms (H1).

To measure hypothetical moral behavior as our dependent variable, we proposed a set of dilemmas. A dilemma, in the broadest sense, is a situation requiring a choice between two equally undesirable or unsatisfactory options. In the literature, the most commonly used dilemmas are those based on the so-called “trolley problem” (Greene et al., 2001, 2004; Moore et al., 2008). These scenarios prompt participants to make moral decisions involving life and death.

Although studies that use these and other moral dilemmas have provided many important insights, many scholars have noted that such scenarios are quite extreme and unrealistic. More broadly, moral dilemmas can arise in situations where it is clear what ought to be undertaken, but there is temptation or pressure to act otherwise (e.g., when individuals are tempted to prioritize self-interest over the interests of others or groups). These situations can still be considered moral dilemmas—though not in the purest sense of representing a decision between equally weighted moral values. These are referred to as false moral dilemmas (Brinkmann, 2005; Kidder, 1995; Kvalnes, 2019; Maclagan, 2003). The false moral dilemmas used in this study involve realistic situations in which self-interest conflicts with the interests of others or groups.

Lastly, we controlled for socio-demographic variables and other factors that may be associated with hypothetical moral behavior, treating some of them as covariates. First, gender is related to different responses to moral dilemmas (e.g., Fumagalli et al., 2010). These authors found that men tend to embrace consequentialist judgment significantly more than women, but only in the case of personal moral dilemmas. Moral judgments can also vary by age (e.g., Friesen, 2019). Since morality may function as an identity marker (e.g., Ellemers et al., 2013), hypothetical moral behavior may differ between individuals with high versus low levels of in-group identity importance. In addition, we control for political orientation, which has been shown to influence moral judgments and behaviors (e.g., Cavazza & Mucchi-Faina, 2008; Kaikati et al., 2017).

## 2. Materials and Methods (Study 1)

### 2.1. Participants

The research involved 184 participants (113 females and 71 males) who completed an anonymous questionnaire. A power analysis (G*Power 3; Faul et al., 2009) indicated that, with a threshold probability of 0.05 and an expected effect size of r = 0.20 (equivalent to an incremental f2 of 0.0416 in multiple regression), the sample size provided approximately 79.08% power (80% power would be achieved with a sample size of 191). Thus, the power can be considered fairly adequate to detect significant associations reflecting the expected effect. Participants’ ages ranged from 18 to 52 years (M = 21.04, SD = 3.70).

### 2.2. Procedure

Participants in this study were recruited from students enrolled in an introductory Social Psychology course at the Faculty of Medicine and Psychology, Sapienza—University of Rome. They were asked to distribute the questionnaire to their friends and acquaintances and, once completed, to return it to the researcher. In return, they received course credit. Participation was voluntary and anonymous. Written informed consent was obtained from all the participants included in this research. The research was approved by the Ethical Research Committee of the authors’ department.

### 2.3. Materials

The questionnaire included several socio-demographic variables (age, gender, place of residence, citizenship, and political orientation, measured on a 7-point scale from 1 = extreme left to 7 = extreme right). Regarding political orientation, 76 participants identified as left-wing or center-left, while the remaining 108 identified as right-wing or center-right. We also asked participants to rate the importance of Italian identity to them on a 6-point scale (1 = not at all important; 6 = extremely important) (importance of in-group identity). The questionnaire included the following scales, presented in this order:

The Moral Foundations Questionnaire (MFQ): Moral foundations were assessed using the scale developed by Graham et al. (2009), which has been validated in Italy (Bobbio et al., 2011). The original questionnaire includes two dimensions: relevance and judgment. According to the authors, the relevance scale examines the explicit theories people hold about what is morally relevant to them, while the judgment scale investigates the extent to which the five moral foundations are concretely used in moral judgments. In this study, to keep the questionnaire as concise as possible, we used only the judgment scale, asking participants to rate their degree of agreement on a 6-point scale. For example: “Compassion for those who suffer is the most important virtue of all” and “People should be loyal to their family members, even if they have done something wrong.” The confirmatory factor analyses (CFAs) showed unsatisfactory fits for both the five-factor and two-factor models (five-factor solution: CFI: 0.722, TLI: 0.635, RMSEA: 0.100, SRMR: 0.092; two-factor solution: CFI: 0.692, TLI: 0.637, RMSEA: 0.100, SRMR: 0.094). Given the mediocre fit of both models, we opted for the two-factor model as a more parsimonious representation of the broader five basic moral foundations. Because both models showed a poor fit, we prioritized simplicity in terms of factor structure. We created two indexes: individualizing (Cronbach’s *α* = 0.65) and binding (*α* = 0.70). As noted in the literature, low reliability is partly due to the MFQ’s broad scope in measuring various moral foundations, which is a common finding in MFQ research (e.g., Federico et al., 2013; Graham et al., 2011).

Set of Moral Dilemmas: For this study, five scenarios were formulated to measure hypothetical moral behavior in real-life situations. Participants were asked to assess the probability (0–100%) of acting morally if they encountered such situations. An exploratory factor analysis was conducted to individuate an underlying factorial structure. The analysis revealed a one-factor structure that explained 46.07% of the variance. This structure demonstrates a good fit also in the confirmatory factor analysis (CFA). The chi-square test was χ2(5) = 10.05, suggesting an adequate fit. However, the chi-square test is sensitive to sample size. The CFI was 0.97 and TLI was 0.94, with both indicating a strong fit. The RMSEA was 0.07 with a confidence interval of [0, 0.14], suggesting a reasonable fit. The SRMR was 0.04, supporting a good fit, as values below 0.08 are typically desirable. We created an index by averaging the responses across five items (*M* = 73.17; *SD* = 19.59; Cronbach’s *α* = 0.68). Higher values indicate a higher probability of hypothetical moral behavior.

1. One evening, you damage someone’s car in an isolated area.
  (a) I would leave my phone number
  (b) I would run away since no one had seen the incident.
2. You need to fill out the form for your tax declaration.
  (a) I would declare the expenses correctly and accurately.
  (b) I would deduct expenses to which I am not entitled.
3. You are looking for a house to rent.
  (a) I would look for a house with a regular contract.
  (b) I would accept renting a house without a contract, even though I know the owner does not pay taxes in this way.
4. You need to move out of your flat and ask an agency to assist you.
  (a) I would pay and ask for a receipt.
  (b) I would pay less without a receipt, even though I know the owner does not pay taxes this way.
5. You have an appointment with a doctor.
  (a) I would pay for the visit and ask for a receipt.
  (b) I would pay less without asking for a receipt.

Perception of the In-group’s Moral Standards: Participants were asked to evaluate, on a scale from 1 (not at all present) to 6 (very frequently present), the presence of various negative phenomena in Italian society (7 items), such as corruption, theft, tax evasion, stealing public money, prostitution, aggression–violence, and drug trafficking and consumption. These items were intended to reflect the moral standards in society. An exploratory factor analysis was conducted to individuate an underlying factorial structure. A scree plot was used to determine the number of factors. The analysis revealed two factors that explain 53.95% of the variance (42.40% the first, and 11.58% the second). We observed that three items had factor loadings greater than 0.4 on both factors. Therefore, we conducted a confirmatory factor analysis (CFA) to further examine the data. The CFA indicated a very good model fit for a two-factor structure, as reflected in the following indices; additionally, the chi-square test (χ2(7) = 17.58, *p* = 0.17) suggested a moderate discrepancy between the model and the data. However, given that the chi-square test is sensitive to sample size, it is best interpreted alongside other fit indices. The CFI was 0.99, and the TLI was 0.98, with both indicating an excellent fit. The RMSEA and SRMR were both 0.04, further supporting a strong model fit. To create indices, we averaged the responses across all relevant items. The first index included four items (corruption, theft, tax evasion, and stealing public money; Cronbach’s α = 0.79), while the second index comprised three items (prostitution, aggression–violence, and drug trafficking and consumption; Cronbach’s α = 0.71). However, due to the high correlation between these two indices (*r* = 0.54), we decided to merge them into a single index (Cronbach’s α = 0.82). This composite index was labeled Perception of the In-group’s Violation of Moral Standards, with higher scores reflecting a more negative perception of the in-group’s moral standards.

## 3. Results

### 3.1. Descriptive Statistics and Correlations

First, we checked for ceiling or floor effects by examining the skewness and kurtosis values for each variable of interest. Except for age (skewness = 3.94, kurtosis = 19.30), all variables presented acceptable values for both indices (no were less than −2 or greater than 2; see George & Mallery, 2010). Thus, we proceeded to calculate the correlations between the two dimensions of moral foundations, the perception of the in-group’s violation of moral standards, and the choices made in moral dilemmas.

The analysis showed that binding moral foundations were positively correlated with individualizing foundations and moral choices (Table 1).

### 3.2. Multiple Regression Analysis

To test our hypotheses, we ran a regression model using the macro PROCESS of the statistical software SPSS 27 (Model 2). We considered moral choices as the criterion variable and included as predictors binding moral foundations, individualizing moral foundations, the index of the perception of the in-group’s violation of moral standards, and the interactions between these variables. All predictors were standardized before the analysis.

The results of the models are reported in Table 2. The model explained a significant portion of the outcome (R^2^ = 0.07, *F* = 2.74, *p* < 0.02). Specifically, we found a significant and positive effect of the interaction between the perception of the in-group’s violation of moral standards and the binding moral foundations (*β* = 0.17, *p* = 0.04), in line with H1.

**Figure 1:**
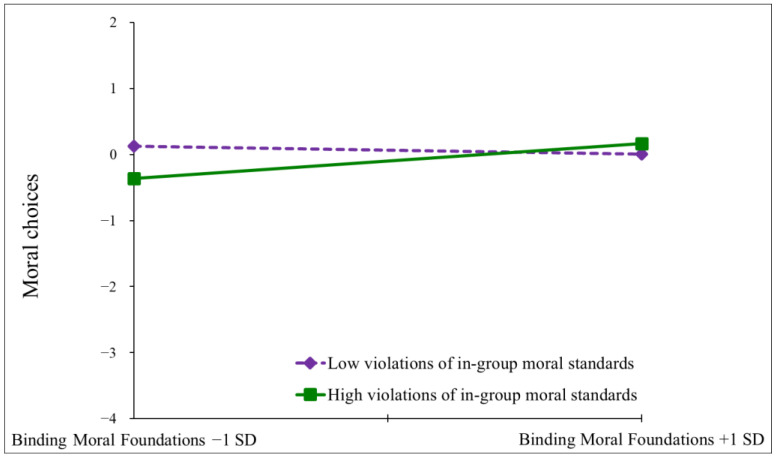
Simple slope analysis of the interaction between violation of in-group’s moral standards and binding foundations on moral behavior (Study 1).

To interpret the significant interaction, we conducted a simple slope analysis (Aiken & West, 1991), computing the effect of binding moral foundations at high (+1 SD) and low (−1 SD) levels on the perception of the in-group’s violation of moral standards. The results revealed that, in the case of low perception of the in-group’s violation of moral standards, the effect of binding moral foundations was non-significant (*β* = −0.10, *t* = −0.82, *p* = 0.41). However, when the perception of the in-group’s violation of moral standards was high, the effect of binding moral foundations was positive and significant (*β* = 0.29, *t* = 0.3.33, *p* < 0.001). For a graphical representation of the simple slopes, see Figure 1.

Overall, the findings suggest that when the in-group is not perceived as violating moral standards there is no difference in moral choices based on the endorsement of binding moral foundations. However, when the in-group is perceived as transgressing its moral standards, the higher the endorsement of binding moral foundations, the more moral choices people make. These findings conform to our predictions for H1.

## 4. Discussion

As described in the introduction, the literature reports contradictory results regarding the role of binding moral foundations in predicting moral attitudes and behavior. Some studies have found that binding is associated with negative attitudes toward out-groups (Baldner & Pierro, 2019; Federico et al., 2016), whereas others have presented a more optimistic view, emphasizing that under certain conditions, endorsing binding moral foundations leads to positive outcomes (e.g., I. H. Smith et al., 2014). We tested whether binding foundations could guide moral choices under specific conditions, namely, when there is a negative perception of the in-group’s moral standards. Binding refers to the foundations of respect for the in-group’s norms, and if people perceive that the in-group is unable to uphold high moral standards then individuals who strongly endorse binding foundations are likely to reinforce their moral standards in situations that require care for both the in-group and society. Our results confirmed these expectations.

Study 2 aims to replicate this pattern of results with an independent group of participants and using an additional measure of moral foundations.

## 5. Materials and Methods (Study 2)

### 5.1. Participants

This study involved 201 participants (80 males and 121 females), who completed an anonymous questionnaire. The sample size provides 80% power to detect a significant effect (*p* < 0.05), assuming r = 0.20 in the population. The age of participants ranged from 15 to 61 years (M = 29.58, SD = 12.05). Regarding political orientation, 45.5% identified as left-wing or center-left, 21.3% identified as right-wing or center-right, and the remaining 33.2% were unsure of their political orientation.

### 5.2. Procedure

Participants for this study were recruited from students attending an introductory course in Social Psychology at the Faculty of Medicine and Psychology, Sapienza—University of Rome, one year after Study 1. They were asked to distribute three questionnaires to their friends and acquaintances, and once completed, to return them to the researcher. In return, they earned course credits. Participation in this study was voluntary and anonymous. Written informed consent was obtained from all the participants included in this research.

### 5.3. Materials

The same socio-demographic variables investigated in Study 1 were included in Study 2 (age, gender, place of residence, and political orientation on a 7-point scale: 1 = left; 7 = right). We asked participants to rate the importance of Italian identity to them on a 6-point scale (1 = not at all; 6 = extremely important) (importance of the in-group identity). In addition, the questionnaire included the following:

Moral Foundations Vignettes (MFVs) (74 items; Clifford et al., 2015). Each vignette is a short description of behavior that violates a particular moral foundation. Participants are asked to evaluate how morally acceptable vs. unacceptable the action is on a 6-point scale (1 = completely unacceptable, 6 = completely acceptable). We excluded scenarios that represented violations of social norms which could be seen as unusual but not morally wrong, and a few scenarios that were not suitable for the Italian context. We reversed the answers so that higher scores indicate a higher moral evaluation. The confirmatory analyses did not produce acceptable fits for either the five-factor model (CFI: 0.730, TLI: 0.645, RMSEA: 0.096, SRMR: 0.091) or the two-factor model (CFI: 0.695, TLI: 0.640, RMSEA: 0.096, SRMR: 0.095). Therefore, we decided to follow the same approach as in Study 1 by creating the two following indexes: individualizing (Cronbach’s *α* = 0.82) and binding (α = 0.83).

A Set of Moral Dilemmas. In this study, we also considered the same five moral dilemmas as in Study 1 and asked participants to assign a percentage (0–100%) to options A and B to evaluate the probability with which they would behave if they found themselves in such a situation. Confirmatory factor analysis (CFA) was conducted to examine if this measure has one latent factor, as in Study 1. The model fit indices suggest an acceptable fit. The chi-square value for the default model was χ2(5) = 6.69, indicating a non-significant discrepancy between the model and the data, and suggesting a reasonable fit. However, chi-square is sensitive to sample size, so other fit indices should be considered. The CFI was 0.97 and the TLI was 0.94, both suggesting a strong model fit. Additionally, the RMSEA was 0.04 with a confidence interval of [0, 0.11], indicating a good fit. The SRMR was 0.05, which falls well within the acceptable range (≤0.08), further supporting a good fit. We computed an index by averaging the responses across five items (*M* = 74.82; *SD* = 15.01; Cronbach’s *α* = 0.65). When high, it indicates a higher probability of a moral choice.

Perception of the In-group’s Moral Standards. The same scale was used as in Study 1. An exploratory factor analysis revealed two factors that explain 55.26% of the variance (41.92% the first, and 13.34% the second). We observed that three items had factor loadings greater than 0.3 on both factors. Therefore, we conducted a confirmatory factor analysis (CFA) to further examine the structure. The model based on two factors demonstrates a good fit, as reflected in the following indices: the chi-square test (χ2(7) = 17.58, *p* < 0.003; CFI) = 0.96, the TLI = 0.94, the RMSEA = 0.05, and the SRMR = 0.05. We created two indexes by averaging the responses across the relevant items. The first index included four items (corruption, theft, tax evasion, and stealing public money; Cronbach’s α = 0.76), while the second index comprised three items (prostitution, aggression–violence, and drug trafficking and consumption; Cronbach’s α = 0.80). However, due to the high correlation between these two indices (*r* = 0.50), we decided to merge them into a single index (Cronbach’s α = 0.82). This composite index was labeled Perception of the In-group’s Violation of Moral Standards, with higher scores reflecting a more negative perception of the in-group’s moral standards.

## 6. Results

### 6.1. Descriptive Statistics and Correlations

As in Study 1, we first examined the values of skewness and kurtosis for the variables of interest. All variables presented acceptable values for both indices (no less than −2 or greater than 2; see George & Mallery, 2010). As shown in Table 3, binding moral foundations were positively associated with individualizing foundations, moral choices, the age of the participants, and right-wing political orientation. Individualizing foundations were positively associated with the perception of the in-group’s violation of moral standards, moral choices, and gender. Moral choices were positively associated with both binding and individualizing moral foundations, and age.

### 6.2. Multiple Regression Analysis

We applied the same regression procedure used in Study 1. This model also explained a significant portion of the variance (R^2^ = 0.18, *F* = 4.52, *p* < 0.001). We found a significant main effect regarding binding moral foundations (*β* = 0.32, *p* = 0.001). In addition, we found a significant and positive effect of the interaction between the perception of the in-group’s violation of moral standards and binding moral foundations (*β* = 0.21, *p* < 0.03), in line with H1 (Table 4). Among the covariates, we found a significant effect regarding age (*β* = 0.18, *p* < 0.01) and political orientation (*β* = −0.18, *p* < 0.01).

**Figure 2:**
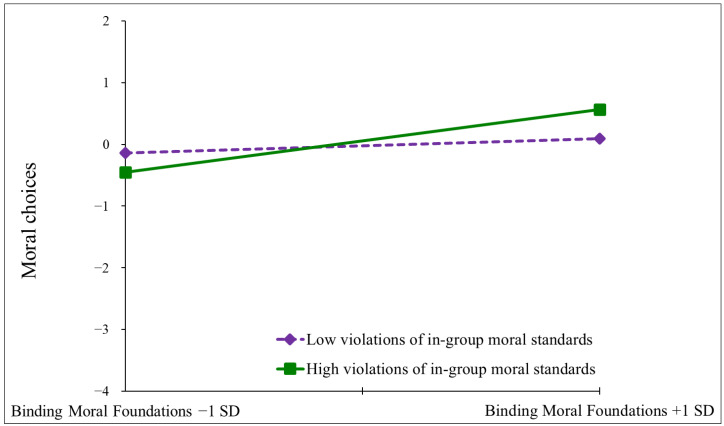
Simple slope analysis of the interaction between violation of in-group’s moral standards and binding foundations on moral behavior (Study 2).

To interpret the significant interaction, we conducted a simple slope analysis (Aiken & West, 1991) using the same procedure as in Study 1. The findings revealed that, in the case of low perception of the in-group’s violation of moral standards the effect of binding moral foundations was non-significant (*β* = 0.10, *t* = 1.02, *p* = 0.31), suggesting that when no particular violation of the in-group’s moral standards is perceived then endorsing binding moral foundations does not affect one’s moral choices. However, in the case of a high perception of the in-group’s violation of moral standards the effect of binding moral foundations was positive and significant (*β* = 0.38, *t* = 4.14, *p* < 0.001), indicating that, in this case, the higher the endorsement of binding moral foundations then the stronger the inclination toward moral choices in the false dilemmas. For a graphical representation of the simple slopes, see Figure 2. Taken together, these findings are consistent with the results from Study 1.

## 7. Discussion

Study 2 replicated the key finding of Study 1 that binding moral foundations are associated with hypothetical moral behavior when the in-group is perceived as problematic in the moral domain. A different measure of moral foundations was used in this study, yielding similar results to those of the previous study. The correlations between the variables are consistent across the two studies, with the exception of some socio-demographic variables.

## 8. General Discussion

According to Haidt and colleagues (Graham et al., 2009, 2013; Haidt, 2013), moral foundations are innate, and people differ in the extent to which they support and use them. However, although these foundations are innate, they can be influenced by various social and cultural factors (Haidt & Graham, 2007; Shweder et al., 1997). As mentioned earlier, several studies have revealed a “dark side” to binding foundations, such as negative attitudes toward out-groups. This raises the question of whether these foundations can predict moral behavior, and if so, under which conditions.

This study showed that binding moral foundations predict choices in moral dilemmas, particularly in interaction with the perception of the in-group’s moral environment. Specifically, people who perceive that the in-group violates general moral standards tend to make more moral choices if they have stronger binding foundations. It seems that binding moral foundations may guide moral behavior when people perceive that their in-group is not aligned with moral standards and norms. In such cases, these foundations could motivate individuals to behave morally to counteract the threat posed by a negative group image, thereby realigning the in-group’s moral standing with its moral standards. This finding is consistent with previous research, which suggests that people who endorse binding foundations may react negatively toward those who deviate from moral norms (Jamieson et al., 2014; Monin, 2007).

Our results highlight a more positive aspect of binding moral foundations and contradict the suggestion made by Kugler et al. (2014) that binding foundations cannot be considered a source of “moral” (in the sense of unambiguously positive) choices and preferences. Our study suggests that people with strong binding foundations may be more likely to behave morally, especially when they perceive that moral standards in their society are being violated. Individuals high in binding foundations are oriented toward respect for the in-group, authority, and social norms. When they observe moral problems or violations of moral standards in society, they strive to protect their own morality and, as a result, tend to behave morally. It is likely that people engage in identity-protective processes in such situations. This finding further confirms that it is impossible to analyze morality and moral behavior without considering the social context and social norms. Therefore, people’s moral behavior depends not only on their moral foundations but also on how they perceive their society, its social norms, and moral standards.

These results align with social identity theory (Tajfel & Turner, 1979). People are motivated to maintain a positive identity and, therefore, to define their groups as moral. The awareness of belonging to dishonest groups can have significant costs in terms of self-esteem (Leach et al., 2007). When the in-group is perceived as immoral, individuals seek to differentiate themselves and present themselves as honest and moral (Brambilla et al., 2011, 2012). Thus, when people perceive society as morally problematic, they experience discomfort, which creates a need to compensate for the in-group’s moral shortcomings by demonstrating more morality in these situations. This is similar to what was found by (Sachdeva et al. 2009), who showed that thinking about immoral aspects of oneself evokes the need to engage in moral behavior. We argue that thinking about immoral aspects of one’s in-group could evoke a similar need to engage in moral behavior. A society composed of individuals who prioritize binding foundations is well equipped to motivate its members to protect the in-group.

The moral dilemmas used in this study involve more realistic situations than those typically found in moral psychology, such as the trolley problem. However, they remain hypothetical scenarios and are subject to social desirability bias. Given that conservatives tend to conform more strongly to norms (Cavazza & Mucchi-Faina, 2008) and that binding moral foundations are endorsed more strongly by conservatives, an alternative explanation for our findings could be that rating negative phenomena makes group norms more salient, encouraging right-wing individuals (conservatives) to select the more socially desirable or conforming option. However, we did not find any significant interaction with political orientation. Furthermore, our results showed that individualizing foundations are also positively correlated with hypothetical moral behavior, suggesting that if social desirability effects were present then they were not limited to binding moral foundations.

As expected, our data confirmed that the relationship between individualizing moral foundations and hypothetical moral behavior is not moderated by the perception of the in-group’s moral standards. Individualizing moral foundations are primarily concerned with protecting the rights and freedoms of individuals and, as such, may represent a form of personal moral identity, regardless of the moral standards of the in-group.

### Limitations and Future Directions

The present studies have some limitations. One limitation is that our data are correlational, meaning we cannot identify the causal mechanisms underlying the findings. Experimental designs which induce a sense of in-group violation of moral standards versus no violation are recommended to identify the causal mechanisms at play.

Second, our study introduced a new scale to measure the perception of negative phenomena in society. Future studies could use alternative measures to assess this issue. For instance, it would be valuable to measure the extent to which participants perceive different phenomena as problems or violations of moral standards in society, in addition to their frequency.

Third, our study was based on a relatively small number of scenarios, most of which focused on various forms of tax evasion, which limits the generalizability of the findings to other contexts. Future research should include additional and more varied moral dilemmas. Additionally, regarding the dependent variable, it is possible that our results are specific to hypothetical moral behaviors that align with in-group binding foundations but do not apply to behaviors aligned with individualizing foundations, such as protecting vulnerable people from harm or making accommodations for disadvantaged individuals. Future studies should explore different operationalizations of the dependent variable that correspond to the types of moral actions most relevant to those who emphasize either binding or individualizing foundations.

Fourth, future studies should create conditions that allow for the testing of real behavior, not just descriptions of hypothetical actions and behaviors. Previous research has found that individualizing moral foundations predict prosocial behavior in economic games, particularly toward strangers (Clark et al., 2017). Future studies could investigate whether this pattern is reversed for those who endorse binding moral foundations when they perceive a group-level threat. Thus, future research might consider using social games such as the dictator game, or examining charitable donations, as dependent variables.

Fifth, our findings showed poor fit indices for both a five-factor and a two-factor model, consistent with previous studies (e.g., Davies et al., 2014; Graham et al., 2011). We agree with scholars who question the empirical and theoretical models of moral foundations, citing flaws in the available measures, the lack of a solid empirical factorial structure, and concerns about the generalizability of findings to other cultures (e.g., Harper & Rhodes, 2021). For these reasons, we believe further research is needed to directly address these controversies. Nevertheless, given the ongoing doubts about both the two-factor and five-factor models in the literature, and until a better model emerges, we believe the distinction between individualizing and binding foundations remains the best approach, prioritizing parsimony over complexity.

Last but not least, as noted by Haidt and his collaborators (see also Atari et al., 2022), the interrelations between the foundations should be considered culture-dependent. Our study was conducted only in Italy with a homogenous group of participants, so generalizability beyond this national context remains unproven. Evidence from surveys across a wide range of nations will be necessary to address this issue.

## 9. Conclusions

This study demonstrates that binding moral foundations, while often viewed as having a “dark side”, can promote moral behavior under specific conditions. By examining the interplay between individual moral predispositions, social contexts, and group norms in shaping moral behavior, we can better understand the complexities of moral decision-making and its implications for fostering moral societies. The interaction between moral foundations and societal perceptions underscores the dynamic nature of morality, wherein individual predispositions interact with social and cultural influences to guide behavior. Further research is needed to explore these interactions in diverse cultural and social settings, paving the way for a more comprehensive understanding of morality and its foundational role in human behavior. For example, future studies should explore the nuanced role of political and ideological factors in shaping responses to moral dilemmas.

## Figures and Tables

**Table 1:** Summary statistics and correlations between variables (Study 1).

	M	SD	α	1	2	3	4	5
1. Binding	3.66	0.86	0.70	-				
2. Individualizing	3.99	0.64	0.65	0.38 **	-			
3. In-group violation of moral standards	4.81	0.65	0.82	0.14 *	0.14	-		
4. Moral choices	73.17	19.59	0.68	0.15 **	0.08	−0.05	-	-
5. Importance of in-group identity	3.55	1.32	-	0.24 **	0.09	−0.01	−0.04	

Note. Gender (Male = 1; Female = 2). * *p* < 0.05. ** *p* < 0.01.

**Table 2:** Moral choices in relation to the binding moral foundations, individualizing moral foundations, perception of in-group’s violation of moral standards, and the interactions between them (Study 1).

	Moral Choices		
	*β*	*SE*	*p*
Binding foundations	0.10	0.08	n.s.
Individualizing foundations	0.02	0.08	n.s.
In-group violation of moral standards	−0.08	0.07	n.s.
Binding foundations × In-group violation of moral standards	0.17	0.08	0.05
Individualizing foundations × In-group violation of moral standards	0.05	0.08	n.s.

**Table 3:** Summary statistics and correlations between variables (Study 2).

	M	SD	α	1	2	3	4	5	6	7	8
1. Binding	4.46	0.85	0.83	-							
2. Individualizing	4.91	0.74	0.82	0.72 **	-						
3. In-group violation of moral standards	5.10	0.63	0.82	0.13 *	0.27 **	-					
4. Moral choices	74.82	15.01	0.65	0.29 **	0.24 **	0.11	-	-			
5. Importance of in-group identity	3.97	1.40	-	0.07	−0.07	−0.03	−0.01	-			
6. Age	29.58	12.05	-	0.19 **	0.08	−0.03	0.22 **	0.28 **	-		
7. Gender	-	-	-	0.10	0.19 **	0.23 **	0.09	−0.10	−0.07	-	
8. Political orientation	3.63	1.34	-	0.18 *	0.01	−0.03	−0.13	0.19 *	0.09	−0.22 **	-

Note. Gender (Male 1; Female 2). * *p* < 0.05. ** *p* < 0.01.

**Table 4:** Moral choices in relation to the binding moral foundations, individualizing moral foundations, perception of in-group’s violation of moral standards, and the interactions between them (Study 2).

	Moral Choices		
	*β*	*SE*	*p*
Gender	0.01	0.07	n.s.
Age	0.18	0.07	0.01
Importance of in-group identity	−0.04	0.07	n.s.
Political orientation	−0.18	0.07	0.01
Binding foundations	0.31	0.10	<0.001
Individualizing foundations	−0.04	0.10	n.s.
In-group violation of moral standards	0.04	0.07	n.s.
Binding foundations × In-group violation of moral standards	0.21	0.09	0.03
Individualizing foundations × In-group violation of moral standards	−0.16	0.09	n.s.

## Data Availability

The datasets are available from the repository: https://osf.io/2kywd/?view_only=16852cec8d9e45ee9381b26423de5b6f (accessed on 20 February 2025).

## References

[B1-behavsci-15-00265] Aiken L., West S. (1991). Multiple regression: Testing and interpreting interactions.

[B2-behavsci-15-00265] Atari M., Haidt J., Graham J., Koleva S., Stevens S. T., Dehghani M. (2022). Morality beyond the WEIRD: How the nomological network of morality varies across cultures. Journal of Personality and Social Psychology.

[B3-behavsci-15-00265] Baldner C., Pierro A. (2019). Motivated prejudice: The effect of need for closure on anti-immigrant attitudes in the United States and Italy and the mediating role of binding moral foundations. International Journal of Intercultural Relations.

[B4-behavsci-15-00265] Bar-Tal D. (2000). Shared beliefs in a society: Social psychological analysis.

[B5-behavsci-15-00265] Bobbio A., Nencini A., Sarrica M. (2011). Il moral foundation questionnaire: Analisi della struttura fattoriale della versione italiana. Giornale di Psicologia.

[B6-behavsci-15-00265] Brambilla M., Rusconi P., Sacchi S., Cherubini P. (2011). Looking for honesty: The primary role of morality (vs. sociability and competence) in information gathering. European Journal of Social Psychology.

[B7-behavsci-15-00265] Brambilla M., Sacchi S., Rusconi P., Cherubini P., Yzerbyt V. Y. (2012). You want to give a good impression? Be honest! Moral traits dominate group impression formation. British Journal of Social Psychology.

[B8-behavsci-15-00265] Brinkmann J. (2005). Understanding insurance customer dishonesty: Outline of a situational approach. Journal of Business Ethics.

[B9-behavsci-15-00265] Cavazza N., Mucchi-Faina A. (2008). Me, us, or them: Who is more conformist? Perception of conformity and political orientation. The Journal of Social Psychology.

[B10-behavsci-15-00265] Clark C. B., Swails J. A., Pontinen H. M., Bowerman S. E., Kriz K. A., Hendricks P. S. (2017). A behavioral economic assessment of individualizing versus binding moral foundations. Personality and Individual Differences.

[B11-behavsci-15-00265] Clifford S., Iyengar V., Cabeza R., Sinnott-Armstrong W. (2015). Moral foundations vignettes: A standardized stimulus database of scenarios based on moral foundations theory. Behavior Research Methods.

[B12-behavsci-15-00265] Davies C. L., Sibley C. G., Liu J. H. (2014). Confirmatory factor analysis of the Moral Foundations Questionnaire: Independent scale validation in a New Zealand sample. Social Psychology.

[B13-behavsci-15-00265] Ellemers N., Pagliaro S., Barreto M. (2013). Morality and behavioural regulation in groups: A social identity approach. European Review of Social Psychology.

[B14-behavsci-15-00265] Ellemers N., Pagliaro S., Barreto M., Leach C. W. (2008). Is it better to be moral than smart? The effects of morality and competence norms on the decision to work at group status improvement. Journal of Personality and Social Psychology.

[B15-behavsci-15-00265] Ellemers N., van den Bos K. (2012). Morality in groups: On the social-regulatory functions of right and wrong. Social and Personality Psychology Compass.

[B16-behavsci-15-00265] Faul F., Erdfelder E., Buchner A., Lang A. G. (2009). Statistical power analyses using G*Power 3.1: Tests for correlation and regression analyses. Behavior Research Methods.

[B17-behavsci-15-00265] Federico C. M., Ekstrom P., Tagar M. R., Williams A. L. (2016). Epistemic motivation and the structure of moral intuition: Dispositional need for closure as a predictor of individualizing and binding morality. European Journal of Personality.

[B18-behavsci-15-00265] Federico C. M., Weber C. R., Ergun D., Hunt C. (2013). Mapping the connections between politics and morality: The multiple sociopolitical orientations involved in moral intuition. Political Psychology.

[B19-behavsci-15-00265] Friesen A. (2019). Generational change? The effects of family, age, and time on moral foundations. The Forum.

[B20-behavsci-15-00265] Fumagalli M., Ferrucci R., Mameli F., Marceglia S., Mrakic-Sposta S., Zago S., Lucchiari C., Consonni D., Nordio F., Pravettoni G., Cappa S., Priori A. (2010). Gender-related differences in moral judgments. Cognitive Processing.

[B21-behavsci-15-00265] George D., Mallery M. (2010). SPSS for windows step by step: A simple guide and reference, 17.0 update.

[B22-behavsci-15-00265] Graham J., Haidt J., Koleva S., Motyl M., Iyer R., Wojcik S. P., Ditto P. H. (2013). Moral foundations theory: The pragmatic validity of moral pluralism. Advances in Experimental Social Psychology.

[B23-behavsci-15-00265] Graham J., Haidt J., Nosek B. A. (2008). The moral foundations questionnaire.

[B24-behavsci-15-00265] Graham J., Haidt J., Nosek B. A. (2009). Liberals and conservatives rely on different sets of moral foundations. Journal of Personality and Social Psychology.

[B25-behavsci-15-00265] Graham J., Nosek B. A., Haidt J., Iyer R., Koleva S., Ditto P. H. (2011). Mapping the moral domain. Journal of Personality and Social Psychology.

[B26-behavsci-15-00265] Gray K., Keeney J. E. (2015). Disconfirming moral foundations theory on its own terms: Reply to Graham (2015). Social Psychological and Personality Science.

[B27-behavsci-15-00265] Greene J. D., Nystrom L. E., Engell A. D., Darley J. M., Cohen J. D. (2004). The neural bases of cognitive conflict and control in moral judgment. Neuron.

[B28-behavsci-15-00265] Greene J. D., Sommerville R. B., Nystrom L. E., Darley J. M., Cohen J. D. (2001). An fMRI investigation of emotional engagement in moral judgment. Science.

[B29-behavsci-15-00265] Haidt J. (2007). The new synthesis in moral psychology. Science.

[B31-behavsci-15-00265] Haidt J. (2008). Morality. Perspectives on Psychological Science.

[B32-behavsci-15-00265] Haidt J. (2013). Moral psychology for the twenty-first century. Journal of Moral Education.

[B30-behavsci-15-00265] Haidt J. (2016). When and why nationalism beats globalism. Policy: A Journal of Public Policy and Ideas.

[B33-behavsci-15-00265] Haidt J., Graham J. (2007). When morality opposes justice: Conservatives have moral intuitions that liberals may not recognize. Social Justice Research.

[B34-behavsci-15-00265] Haidt J., Graham J., Joseph C. (2009). Above and below left-right: Ideological narratives and moral foundations. Psychological Inquiry.

[B35-behavsci-15-00265] Haidt J., Joseph C. (2004). Intuitive ethics: How innately prepared intuitions generate culturally variable virtues. Daedalus.

[B36-behavsci-15-00265] Haidt J., Joseph C., Carruthers P., Laurence S., Stich S. (2008). The moral mind: How 5 sets of innate intuitions guide the development of many culture-specific virtues, and perhaps even modules. The innate mind.

[B37-behavsci-15-00265] Harper C. A., Rhodes D. (2021). Reanalysing the factor structure of the moral foundations questionnaire. British Journal of Social Psychology.

[B38-behavsci-15-00265] Jamieson J. P., Valdesolo P., Peters B. J. (2014). Sympathy for the devil? The physiological and psychological effects of being an agent (and target) of dissent during intragroup conflict. Journal of Experimental Social Psychology.

[B39-behavsci-15-00265] Janoff-Bulman R., Carnes N. C. (2013). Surveying the moral landscape: Moral motives and group-based moralities. Personality and Social Psychology Review.

[B40-behavsci-15-00265] Kaikati A. M., Torelli C. J., Winterich K. P., Rodas M. A. (2017). Conforming conservatives: How salient social identities can increase donations. Journal of Consumer Psychology.

[B41-behavsci-15-00265] Kidder R. M. (1995). How good people make tough choices.

[B42-behavsci-15-00265] Kugler M., Jost J. T., Noorbaloochi S. (2014). Another look at moral foundations theory: Do authoritarianism and social dominance orientation explain liberal-conservative differences in “moral” intuitions?. Social Justice Research.

[B43-behavsci-15-00265] Kvalnes Ø. (2019). Moral dilemmas. Moral reasoning at work.

[B44-behavsci-15-00265] Leach C. W., Bilali R., Pagliaro S., Mikulincer M., Shaver P. R., Dovidio J. F., Simpson J. A. (2015). Groups and morality. APA handbooks in psychology. APA handbook of personality and social psychology, Vol. 2. Group processes.

[B45-behavsci-15-00265] Leach C. W., Ellemers N., Barreto M. (2007). Group virtue: The importance of morality (vs. competence and sociability) in the positive evaluation of in-groups. Journal of Personality and Social Psychology.

[B46-behavsci-15-00265] Leach C. W., Vliek M. L. W. (2008). Group membership as a “frame of reference” for interpersonal comparison. Social and Personality Psychology Compass.

[B47-behavsci-15-00265] Lewis G. J., Bates T. (2010). From left to right: How the personality system allows basic traits to influence politics via characteristic moral adaptations. British Journal of Psychology.

[B48-behavsci-15-00265] Lewis G. J., Kanai R., Bates T. C., Rees G. (2012). Moral values are associated with individual differences in regional brain volume. Journal of Cognitive Neurosciences.

[B49-behavsci-15-00265] Maclagan P. (2003). Varieties of moral issue and dilemma: A framework for the analysis of case material in business ethics education. Journal of Business Ethics.

[B50-behavsci-15-00265] Malka A., Osborne D., Soto C. J., Greaves L. M., Sibley C. G., Lelkes Y. (2016). Binding moral foundations and the narrowing of ideological conflict to the traditional morality domain. Personality and Social Psychology Bulletin.

[B51-behavsci-15-00265] Mededović J., Petrović B. (2016). Can there be an immoral morality? Dark personality traits as predictors of Moral foundations. Psihologija.

[B52-behavsci-15-00265] Monin B. (2007). Holier than me? Threatening social comparison in the moral domain. Revue Internationale de Psychologie Sociale.

[B53-behavsci-15-00265] Moore A. B., Clark B. A., Kane M. J. (2008). Who shalt not kill? Individual differences in working memory capacity, executive control, and moral judgment. Psychological Science.

[B54-behavsci-15-00265] Musschenga B. (2013). The promises of moral foundations theory. Journal of Moral Education.

[B55-behavsci-15-00265] Nilsson A., Erlandsson A. (2015). The moral foundations taxonomy: Structural validity and relation to political ideology in Sweden. Personality and Individual Differences.

[B56-behavsci-15-00265] Pagliaro S., Ellemers N., Barreto M. (2011). Sharing moral values: Anticipated in-group respect as a determinant of adherence to morality-based (but not competence-based) group norms. Personality and Social PsychologyBulletin.

[B57-behavsci-15-00265] Pagliaro S., Ellemers. N., Barreto M., Di Cesare C. (2016). The impact of perceived pervasiveness of moral evaluations of the self on motivation to restore a moral reputation. Frontiers in Psychology.

[B58-behavsci-15-00265] Prinz J. (2008). The emotional construction of morals.

[B68-behavsci-15-00265] Sachdeva S., Iliev R., Medin D. L. (2009). Sinning saints and saintly sinners: The paradox of moral self-regulation. Psychological Science.

[B59-behavsci-15-00265] Shweder R. A., Much N. C., Mahapatra M., Park L., Brandt A. M., Rozin P. (1997). The “big three” of morality (autonomy, community, divinity) and the “big three” explanations of suffering. Morality and health.

[B60-behavsci-15-00265] Smith I. H., Aquino K., Koleva S., Graham J. (2014). The moral ties that bind even to out-groups: The interactive effect of moral identity and the binding moral foundations. Psychological Science.

[B61-behavsci-15-00265] Smith K. B., Alford J. R., Hibbing J. R., Martin N. G., Hatemi P. K. (2017). Intuitive ethics and political orientations: Testing moral foundations as a theory of political ideology. American Journal of Political Science.

[B62-behavsci-15-00265] Tajfel H., Turner J. C., Austin W. G., Worchel S. (1979). An integrative theory of intergroup conflict. The social psychology of intergroup relations.

[B63-behavsci-15-00265] Theodorou A., Kosic A. (2021). Need for closure, morality, and prejudice: The relationship between the need for closure, stereotyped in-group and out-group morality, and prejudice toward the out-group. Social Psychology.

[B64-behavsci-15-00265] Wojciszke B. (2005). Morality and competence in person- and self-perception. European Review of Social Psychology.

[B65-behavsci-15-00265] Wojciszke B., Bazinska R., Jaworski M. (1998). On the dominance of moral categories in impression formation. Personality and Social Psychology Bulletin.

[B66-behavsci-15-00265] Wright J. C., Baril G. (2011). The role of cognitive resources in determining our moral intuitions: Are we all liberals at heart?. Journal of Experimental Social Psychology.

[B67-behavsci-15-00265] Yılmaz O., Harma M., Bahçekapılı H. G., Cesur S. (2016). Validation of the moral foundations questionnaire in Turkey and its relation to cultural schemas of individualism and collectivism. Personality and Individual Differences.

